# Unveiling the predictive power: a comprehensive study of machine learning model for anticipating chronic kidney disease

**DOI:** 10.3389/frai.2023.1339988

**Published:** 2024-01-05

**Authors:** Nitasha Khan, Muhammad Amir Raza, Nayyar Hussain Mirjat, Neelam Balouch, Ghulam Abbas, Amr Yousef, Ezzeddine Touti

**Affiliations:** ^1^Department of Electrical Engineering, Nazeer Hussain University, Karachi, Pakistan; ^2^Department of Electrical Engineering, Mehran University of Engineering and Technology, Khairpur Mirs, Sindh, Pakistan; ^3^Department of Electrical Engineering, Mehran University of Engineering and Technology, Jamshoro, Sindh, Pakistan; ^4^Department of Zoology, Shah Abdul Latif University Khairpur Mirs, Khairpur Mirs, Pakistan; ^5^School of Electrical Engineering, Southeast University, Nanjing, China; ^6^Electrical Engineering Department, University of Business and Technology, Jeddah, Saudi Arabia; ^7^Engineering Mathematics Department, Alexandria University, Alexandria, Egypt; ^8^Department of Electrical Engineering, College of Engineering, Northern Border University, Arar, Saudi Arabia

**Keywords:** forecasting, public health, medicine, deep learning, machine learning

## Abstract

In today's modern era, chronic kidney disease stands as a significantly grave ailment that detrimentally impacts human life. This issue is progressively escalating in both developed and developing nations. Precise and timely identification of chronic kidney disease is imperative for the prevention and management of kidney failure. Historical methods of diagnosing chronic kidney disease have often been deemed unreliable on several fronts. To distinguish between healthy individuals and those afflicted by chronic kidney disease, dependable and effective non-invasive techniques such as machine learning models have been adopted. In our ongoing research, we employ various machine learning models, encompassing logistic regression, random forest, decision tree, k-nearest neighbor, and support vector machine utilizing four kernel functions (linear, Laplacian, Bessel, and radial basis kernels), to forecast chronic kidney disease. The dataset used constitutes records from a case-control study involving chronic kidney disease patients in district Buner, Khyber Pakhtunkhwa, Pakistan. For comparative evaluation of the models in terms of classification and accuracy, diverse performance metrics, including accuracy, Brier score, sensitivity, Youden's index, and F1 score, were computed.

## 1 Introduction

Chronic kidney disease (CKD) represents a global public health concern (Levey et al., 2007). The Global Burden of Disease Study in 2010, ranking causes of mortality worldwide from 1990 to 2010, highlighted CKD's rise from the 27th to the 18th position over two decades (Lozano et al., 2012). This escalating CKD epidemic has led to an 82% increase in kidney disease-related years of life lost, a toll akin to diabetes (Rostron et al., 2023). The looming specter of premature death is compounded as most CKD survivors progress to end-stage renal failure. This debilitating condition diminishes life quality and inflicts extensive societal and financial costs. The incidence of renal replacement therapy (RRT) displays significant disparities across nations, ranging from 150 to 400 per million inhabitants (PMI) in developed countries to 50 PMIs or less in developing regions, revealing constrained healthcare resources. Global screening systems are being augmented to curb CKD progression and improve survivability. Consequently, precise knowledge of CKD's prevalence on national and international scales is pivotal in engaging key stakeholders like patients, general practitioners, nephrologists, and funding bodies to devise and enforce effective preventive strategies (Eckardt et al., 2013; Jayasumana et al., 2014; Rapa et al., 2019).

In contemporary times, CKD poses an increasingly grave concern across developed and developing nations. The adoption of unhealthy lifestyles, contributing to diabetes and hypertension, has surged due to urbanization in developing countries. Notably, 15–25% of individuals with diabetes succumb to kidney disease. For instance, in Pakistan, the rapid spread of CKD is attributed to factors such as consumption of substandard food, self-medication, excessive drug use, polluted water, obesity, hypertension, anemia, diabetes, and kidney stones (Imtiaz et al., 2018). In contrast, developed nations like the United States harbor 26 million adults (one in nine) grappling with CKD, who are also at heightened risk for other diseases. Researchers in the USA have devised an eight-point risk factor framework to forecast CKD, encompassing parameters like advanced age, female gender, anemia, diabetes, hypertension, cardiovascular disease, and peripheral vascular disease (Naqvi et al., 2019). Present studies indicate a worldwide CKD prevalence ranging from 5 to 15%, with ~5–10 million annual deaths attributed to CKD (Wang et al., 2023a). As CKD exacerbates with time, early detection and effective interventions offer pragmatic ways to mitigate the mortality rate.

Machine learning (ML) methodologies have emerged as pivotal tools in medical domains for disease identification due to their specific attributes (Elsheikh et al., 2021; Salazar et al., 2021). A study by Khamparia and Pandey (2020) employed the support vector machine (SVM) technique to diagnose CKD, coupled with diverse data reduction methods, including principal component analysis (PCA). Notably, their findings favored the SVM model employing the Gaussian radial basis kernel for superior diagnostic precision and accuracy over competing models. Other researchers have examined kernel functions in random forest models. At the same time, some studies have compared artificial neural networks (ANN) and SVM algorithms to classify and predict varied kidney diseases, with ANN demonstrating the highest accuracy among them. Similarly, statistical and computational intelligence models have been juxtaposed, utilizing class-balanced order for dual classes of non-uniform distribution (Zhao and Zhang, 2008; Wang et al., 2023b). An investigation by Dritsas and Trigka (2022) detailed the identification of suitable dietary plans for CKD patients, utilizing multiple classification methods, with the multi-class decision forest model achieving the highest accuracy (99.17%).

Additionally, supervised classifier techniques, widely employed in diagnosing diverse ailments, have been adopted. Wickramasinghe et al. (2017) used k-nearest neighbor (KNN) and SVM classifiers for CKD dataset prediction, with KNN exhibiting superior performance compared to SVM. A comparative analysis by Hsu et al. (2018) assessed ML and classical regression models in which artificial neural networks achieved the highest accuracy (93%). Recent research primarily employs ML models in medical research. Tazin et al. (2016) harnessed various ML techniques, such as ANN, backpropagation, and SVM, for classifying and predicting patients with kidney stone disease, with backpropagation proving the most effective. In parallel, SVM models were compared with naive Bayes (NB) models, revealing SVM's superiority in disease classification (Kavakiotis et al., 2017; Zhang et al., 2018). In this study, diverse ML models were scrutinized for CKD prediction.

This research study undertakes CKD prediction utilizing various ML models, with its primary contributions outlined as follows:

- Incorporation of primary data from CKD patients in district Buner, Kyber Pakhtunkhwa, Pakistan, the machine and deep learning algorithms aimed at effectively distinguishing healthy individuals from those afflicted by CKD. In particular, the pertinent for developing nations.
- Examination of three different training and testing set scenarios: (a) 90% training, 10% testing; (b) 75% training, 25% testing; and (c) 50% training, 50% testing, along with 1,000 simulations per validation scenario to gauge model consistency.
- A Comparison of prominent machine learning models encompassing: XGBoost Classifier (XGC), Neural Network (NN), Gaussian Naive Bayes (GNB), Logistic Regression (LR), Random Forest (RF), Decision Tree (DT), K-Nearest Neighbor (KNN), and Support Vector Machine (SVM) with four kernel functions (linear, Laplacian, Bessel, and radial basis kernels) is performed for CKD prediction.
- Evaluation of model performance through six metrics: accuracy, recall, error rate, Youden's index, specificity, and F1 score to assess the statistical significance of differences in prediction performance across models.

The subsequent sections of this article are structured as follows: Section 2 presents materials and methods, Section 3 covers results and discussion, and Section 4 concludes the study.

## 2 Data collection and research method

**Figure 1:**
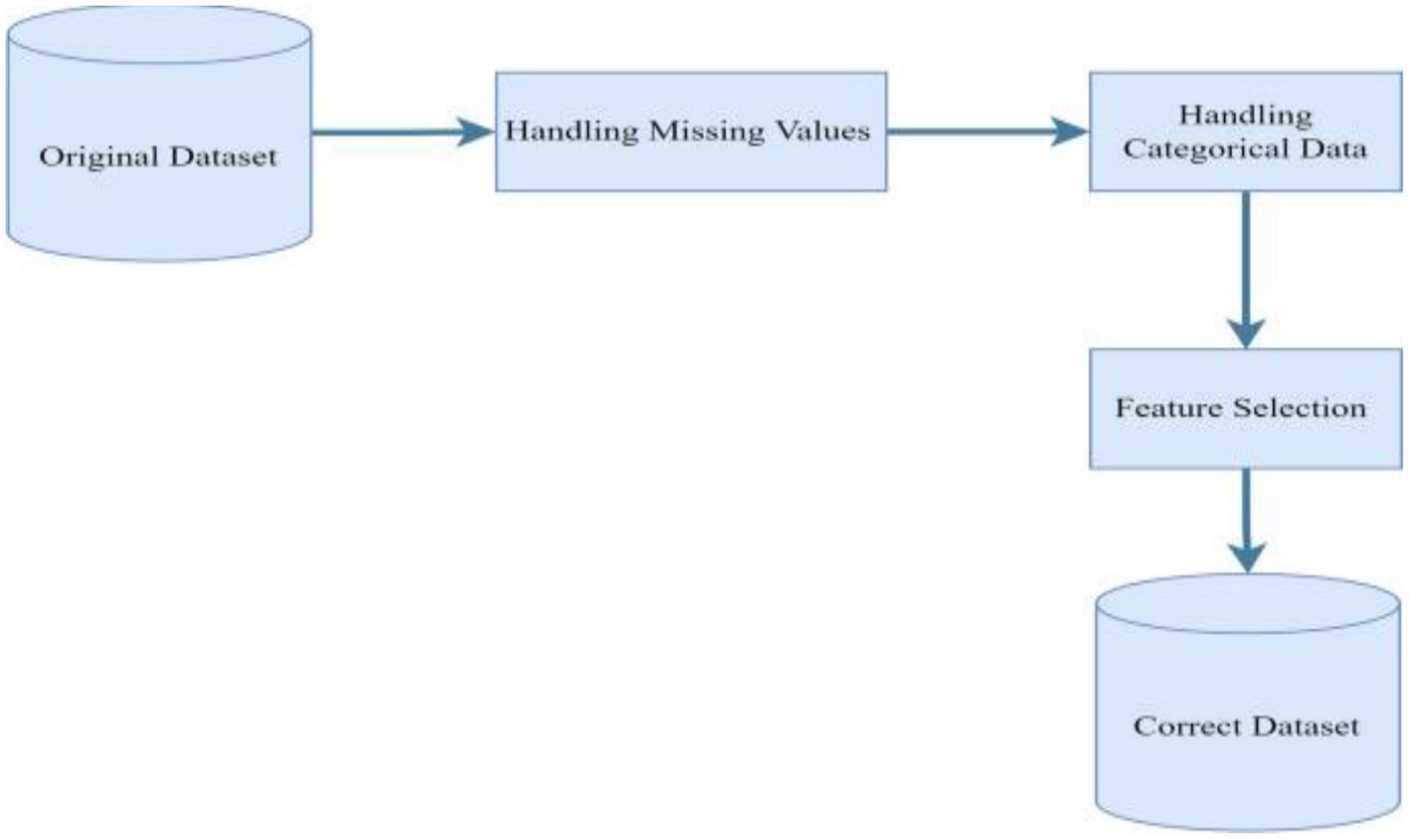
Chronic kidney disease dataset preprocessing steps.

In this section, an in-depth discussion is presented on the selected predictive models, along with a detailed description of the features utilized in the kidney disease dataset from district Buner. Real world data is often inconsistent which can affect the performances of models. Preprocessing the data before it is fed into classifers is vital part of developing machine-learning model. Similarly, the dataset for this study contains missing values that needs to be handled appropriately. It has to also be in a suitable format for modeling. Hence, pre-processing has been conducted as it has been shown in Figure 1.

### 2.1 Feature description

The dataset was collected from the medical complex in Buner, Khyber Pakhtunkhwa, Pakistan. It comprises diagnostic test reports from numerous patients who sought check-ups with nephrologists at the Medical Complex. The dataset encompasses 21 categorical variables, meticulously outlined in Table 1. Data collection spanned from November 2020 to March 2021, with the sample size calculated following the formula stated in Singh et al. (2022).

**Table 1:** Variable report.

**Variable**	**Scale of variables**	**Notation (counts)**	**Label**
Age	Numerical	Years (12–99)	-
Ph specific gravity	Numerical	Mean (5.565) Sd (0.561)	-
Gender	Nominal	Mean (1.016) Sd (0.0052)	-
Urine color	Nominal	Yellow (243) Pale yellow (137)	1
Albumin	Nominal	Trace (227) Nil (153)	1
Glucose	Nominal	Trace (27) Nil (353)	1
Sugar	Nominal	Positive (63) Nil (317)	1
Ketone bodies	Nominal	Trace (64) Not trace (316)	1
Bile pigment	Nominal	Present (64) Absent (316)	1
Urobilinogen	Nominal	Abnormal (38) Normal (342)	1
Blood	Nominal	Positive (62) Negative (318)	1
Mucus thread	Nominal	Present (181) None (199)	1
Calcium oxalate	Nominal	Positive (112) Nil (268)	1
Granular cast	Nominal	Seen (94) Nil (286)	1
Bacteria	Nominal	Seen (123) Not seen (257)	1
Calcium carbonate	Nominal	Found (335) Not found (45)	1
Red cells/RBCs	Nominal	Normal (217) Abnormal (163)	0
Epithelial cells	Nominal	Nil (153) Positive (227)	0
Pus cells/WBCs	Nominal	Normal (166) Abnormal (214)	0
Disease status	Nominal	CKD (240) Not CKD (142)	1
Variable	Scale of variables	Notation (Counts)	Label

WBC, White Blood Cells; RBC, Red Blood Cells; Pale yellow.

The formula used for sample size determination is given by:


(1) $$\begin{array}{l} n=\frac{z^{2}pq}{m} \end{array}$$

Here, *n* represents the sample size, *z* signifies the statistic associated with a certain confidence level, *p* denotes the anticipated prevalence proportion of CKD patients, *q* equals 1 - p, and *m* represents the precision linked with an effect size. For our calculations, it was assumed that 270 patients were afflicted with CKD and 230 were not. Consequently, the anticipated prevalence proportion was computed as *p* = 0.54, *q* = 0.46, *z* = 1.96 (95% confidence interval), and *m* = 0.05. Notably, for expected prevalence proportions falling between 10 and 90%, a 5% precision is recommended (Pourhoseingholi et al., 2013). Plugging in the values of p, z, and m into Equation 1 yielded an approximate sample size of *n* = 382, which was subsequently employed for analysis within this study. Figure 1 illustrates the concept of complete correlation through a heatmap, providing insights into the interrelationships among various categorical attributes within the context of kidney chronic disease analysis. The heatmap showcases a range of nominal variables associated with the disease, highlighting the extent to which these variables exhibit shared patterns or behaviors. Brighter color tones in the heatmap signify stronger correlations between pairs of attributes, shedding light on factors that tend to change in tandem. Conversely, darker color tones indicate weaker or negative correlations, indicating attributes that may vary independently.

**Figure 2:**
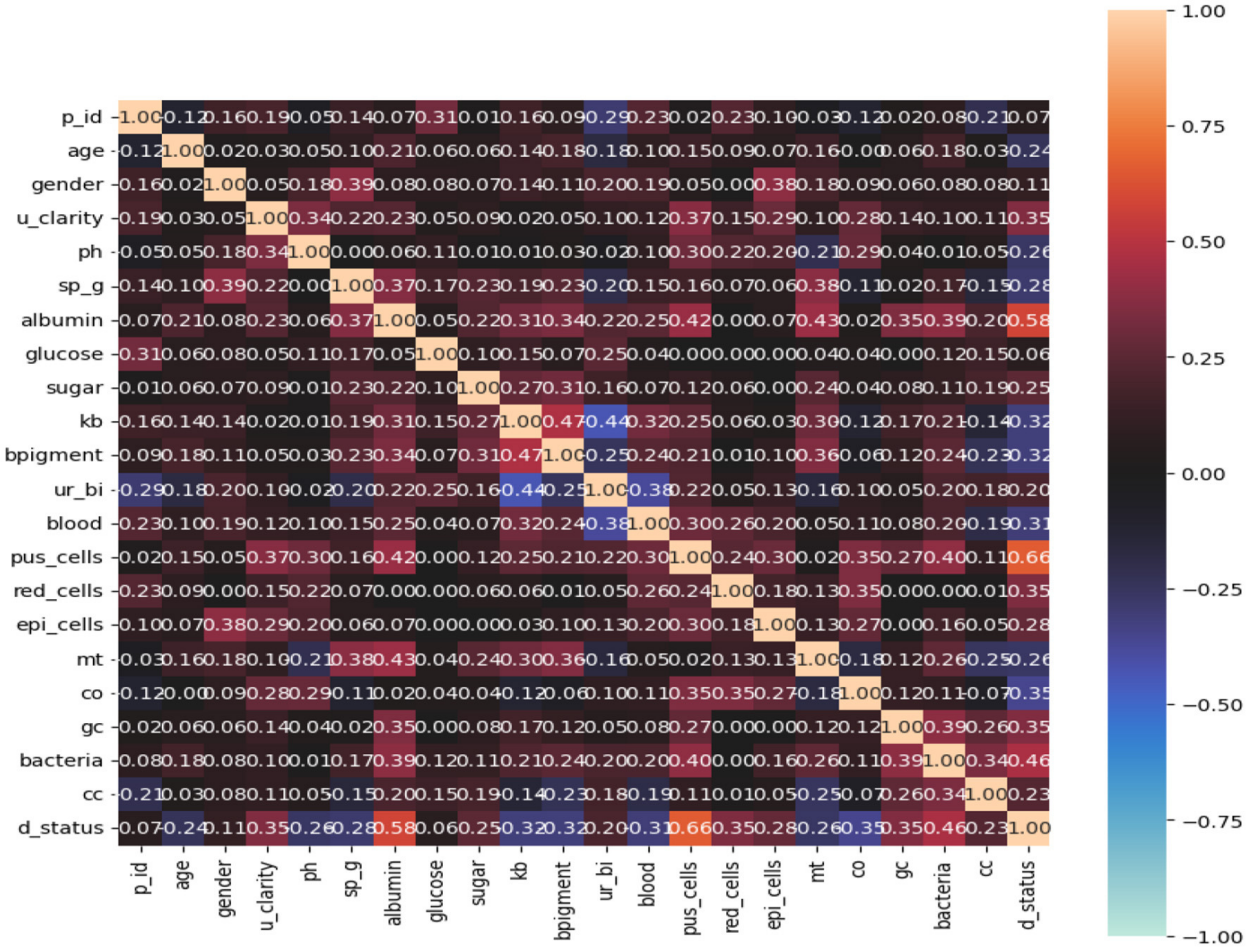
Visual representation to the disease's characteristics and progression.

This visualization is invaluable for discerning patterns and clusters of attributes that might have a collective impact on chronic kidney disease. By understanding these associations, medical professionals, researchers, and policymakers gain a deeper understanding of how different categorical factors interact within the complex landscape of the disease. Such insights inform targeted interventions, personalized treatment approaches, and strategies for managing chronic kidney disease effectively. Overall, Figure 2 serves as a visual representation that aids in comprehending the intricate relationships that contribute to the disease's characteristics and progression.

### 2.2 Machine learning model specifications

In this section, we delve into the specific machine learning predictive models harnessed for this study. The chosen models encompass various algorithms, each designed to offer unique strengths in capturing patterns, making predictions, and classifying data. To facilitate data analysis and model implementation, the statistical software Anaconda was employed on various variables shown in Table 1. Anaconda provides a comprehensive suite of tools and libraries for data manipulation, analysis, and machine learning, offering a cohesive environment for researchers to conduct their experiments effectively and efficiently. In the subsequent sections, we delve into the specifics of the model training, validation, and evaluation processes, shedding light on the strategies adopted to fine-tune parameters and achieve optimal model performance. The following models were utilized in the analysis.

#### 2.2.1 Logistic regression (LR)

LR is a dominant and well-established supervised classification technique (Naing et al., 2006). It extends the regression family and is used to model binary variables indicating the presence or absence of an event. It can also be generalized to a multiple variable model known as multiple logistic regression (MLR). The mathematical representation of MLR is as follows:


(2) $$\begin{array}{l} P_{r}\left(Z\right)=\frac{\exp\left(a_{0}+a_{1}z_{1}+a_{2}z_{2+\ldots +}a_{k}z_{k}\right)}{1+\exp\left(a_{0}+a_{1}z_{1}+a_{2}z_{2+\ldots +}a_{k}z_{k}\right)} \end{array}$$

Here, Z = (*z*_1_,*z*_2_, …*z*_*k*_) comprises k predictors, where *k* = 21 in our case, as elaborated in the previous section. The unknown parameters are estimated using the Maximum Likelihood Estimation (MLE) method.

LR is a fundamental and widely employed method within supervised machine learning, primarily applied for binary classification tasks. Unlike its name suggests, Logistic Regression is used for classification, not regression. This algorithm is characterized by its ability to model the probability of an instance belonging to a particular class. It is particularly suitable when the dependent variable is categorical, and the goal is to predict the likelihood of an event occurrence. Logistic regression employs the logistic function, also known as the sigmoid function, to transform linear combinations of input features into values between 0 and 1. These transformed values represent the probability that a given instance belongs to the positive class. The model's coefficients, derived through iterative optimization techniques like gradient descent, determine the impact of each feature on the predicted probability. A threshold value is chosen to make predictions; instances with predicted probabilities above the threshold are classified into the positive class, while those below the threshold are classified into the negative class. Logistic regression's adaptability to complex relationships in the data makes it versatile, and it can be extended to multi-class classification through techniques like One-vs-Rest. Despite its simplicity and interpretability, the algorithm has limitations, including sensitivity to irrelevant features, a linear decision boundary, and potential overfitting when dealing with high-dimensional data. In essence, while Logistic Regression serves as an essential tool for binary classification tasks, researchers must be cognizant of its assumptions, strengths, and limitations when applying it to diverse datasets and scenarios.

#### 2.2.2 K-nearest neighbor (KNN)

The KNN algorithm stands as one of the foundational techniques in supervised machine learning, anchored firmly on the principle of proximity-based decision-making. Unlike many algorithms that rely on explicit model generation, KNN predicates its predictions on the similarity between data points in the feature space. Precisely, a new data point's classification is ascertained by inspecting its closeness to “K” data points from the training dataset (Sinha and Sinha, 2015). The choice of “K” holds paramount importance; larger values tend to produce smoother decision boundaries, reducing the risk of overfitting, while smaller values make the model more receptive to local nuances in the data. To determine the “nearness” of data points, a standard distance metric, typically the Euclidean distance, is employed. In classification, the algorithm conducts a majority vote among the k-nearest neighbors. At the same time, for regression tasks, it predicts the outcome based on the average or potentially the weighted average of the k-nearest data points' target values. One of KNN's distinct advantages is its ability to adapt decision boundaries according to the local data density, enabling it to handle both linear and non-linear distributions adeptly. Nonetheless, while the adaptability and simplicity of KNN render it appealing, researchers must exercise caution: the algorithm's performance can be sensitive to data density and, when exposed to large datasets or high-dimensional spaces, can succumb to computational inefficiencies and the so-called “curse of dimensionality.” In conclusion, while KNN offers a robust mechanism to leverage data proximity for predictions, careful parameter optimization and consideration of the dataset's characteristics are crucial for its successful application.

#### 2.2.3 Support vector machine (SVM)

SVM models are versatile tools for classifying linear and non-linear data. The process involves mapping each data point into a variable space with k dimensions. A hyperplane is then used to segregate data items into two distinct classes while maximizing the margin between classes and minimizing classification errors (Joachims, 1998). The margin for a class refers to the distance between the nearest instance and the decision hyperplane associated with that class. SVM models incorporate various kernel functions to transform input data into the required format. Notably, four kernel types—linear, Laplacian, Bessel, and radial—are adopted as foundational elements.

Linear Kernel: The linear kernel function calculates the inner product between two points within a suitable feature space:


(3) $$\begin{array}{l} K\left(z_{m},z_{n}\right)=\left(z_{m}.z_{n}+1\right) \end{array}$$

Laplacian Kernel: The Laplacian kernel function is akin to the exponential kernel, with reduced sensitivity to changes in the sigma parameter:


(4) $$\begin{array}{l} K\left(z_{m},z_{n}\right)=e\frac{-||z_{m}.z_{n}||}{a} \end{array}$$

Bessel Kernel: The Bessel kernel, prevalent in the theory of fractional smoothness kernel function spaces, is represented as:


(5) $$\begin{array}{l} K\left(z_{m},z_{n}\right)=\frac{Bv+1\;||z_{m}.z_{n}||}{{\left||z_{m}.z_{n}|\right|}^{n\left(v+1\right)}} \end{array}$$

Radial Basis Kernel: The radial basis kernel, or RBF, is commonly used in SVM models for various kernelized learning applications:


(6) $$\begin{array}{l} K\left(z_{m},z_{n}\right)=e^{\left(-a||z_{m}.z_{n}|{|}^{2}\right)} \end{array}$$

#### 2.2.4 Decision tree (DT)

DT embodies a tree-like structure where nodes correspond to features, branches signify decision rules, and leaves indicate categorical or continuous outcomes. The central concept behind DT involves creating a tree-like pattern for the entire dataset. Each leaf processes a single outcome to minimize errors. DT divides observations into branches to enhance prediction accuracy. The algorithm identifies variables and their cutoff points, segmenting input observations into subsets using techniques like information gain, Gini index, chi-squared test, etc. The splitting process recurs until the complete tree is constructed. The goal of splitting algorithms is to identify variable thresholds that enhance homogeneity in sample outputs. Decision trees are non-parametric and partition data using mechanisms to uncover potential feature values (Criminisi et al., 2012). Overfitting concerns are addressed by adjusting hyperparameters like maximum depth and maximum leaf nodes. In this study, hyperparameters are tuned iteratively with maximum depths of 5, 10, 15, 20, 25, and 30.

#### 2.2.5 Random forest (RF)

RF is a supervised machine learning algorithm that assembles a “forest” of decision trees, introducing an element of randomness. Deep decision trees often lead to overfitting due to excessive specialization, causing significant classification outcome deviations for minor input variations. RF combats this by training different decision trees using distinct training datasets. For classifying a new sample, the input vector is processed through each decision tree. Subsequently, each decision tree evaluates a different part of the input vector, yielding a classified outcome. The final classification is determined based on the majority “votes” in discrete classification cases or the average of all trees in numeric classification cases. By considering multiple decision trees, the random forest algorithm mitigates variations stemming from relying on a single tree for the dataset (Tripepi et al., 2008; Tyralis et al., 2019).

#### 2.2.6 Neural networks (NN)

NN stand as a cornerstone in intense learning stages due to their remarkable ability to capture complex patterns and representations in data. Modeled after the human brain's structure, a neural network comprises layers of interconnected nodes, or neurons, each processing and transforming input data. The input layer receives raw data, traversing through one or more hidden layers, with each layer applying weighted transformations through activation functions. These transformations progressively extract and combine features from the input. The final output layer produces predictions or classifications. The network's architecture, including the number of hidden layers, neurons per layer, and connectivity patterns, determines its capacity to capture intricate relationships within the data. Neural networks excel in tasks such as image and speech recognition, language processing, and complex data analysis. Learning within a neural network occurs through backpropagation, where prediction errors are iteratively fed back to adjust the weights of connections, optimizing the model's performance. However, the successful application of neural networks demands careful parameter tuning, substantial computational resources, and sufficient labeled data for training. Their complexity can lead to overfitting if not appropriately managed, necessitating techniques like regularization and dropout. In summary, neural networks offer a powerful tool for tackling intricate data challenges, yet their potential comes with considerations that researchers must navigate for optimal results (Almansour et al., 2019).

#### 2.2.7 XGBoost classifier (XGC)

The XGC is an indispensable instrument in the machine learning toolkit, primarily due to its efficacy in structured or tabular data classification challenges. An acronym for Extreme Gradient Boosting, XGC (Ogunleye and Wang, 2019) operates on the foundation of the gradient boosting framework. It builds an ensemble of decision trees sequentially, with each tree trying to correct the errors of its predecessor. What sets XGC apart is its ability to optimize both the tree structure and leaf weights using advanced regularization techniques. The model leverages the gradient information of the loss function, making it adaptable to differentiable loss functions, thereby supporting regression, classification, and ranking tasks. One of its notable strengths is its efficient handling of missing data and built-in capability for feature selection. In the realm of computational efficiency, XGC shines by utilizing parallel processing for tree construction and by its capability to cross-validate at each iteration, selecting the best tree structure. Furthermore, it offers regularization parameters to prevent overfitting, which, combined with its scalability, renders it a popular choice in various machine-learning competitions, including Kaggle. However, while XGC boasts versatility and power, understanding its hyperparameters is essential for optimal results. A well-tuned XGC boost model can outperform other algorithms, but care must be taken to ensure its complexity does not overshadow interpretability. In essence, XGC provides a harmonious blend of computational performance and predictive prowess, establishing it as a formidable player in ensemble machine-learning methodologies.

#### 2.2.8 Gaussian Naive Bayes (GNB)

The GNB classifier emerges as a foundational pillar in machine learning and is incredibly esteemed for its simplicity, speed, and suitability for high-dimensional datasets. Rooted in Bayes' theorem, GNB is a probabilistic classifier that presumes each feature is typically distributed and makes a “naive” assumption of independence among features. This means it assumes that the presence of a particular feature in a class is unrelated to the presence of any other feature. Despite its simplicity, GNB operates by calculating the probability of a particular event based on prior knowledge of conditions related to that event. In practice, for each class, it computes the mean and variance of the features in the training data. When making predictions, it uses these statistics to determine the likelihood of a particular data point belonging to each class. Because of its probabilistic nature, GNB (Rabby et al., 2019) has the advantage of naturally handling missing values and providing calibrated probabilities for predictions. While the naive feature independence assumption might seem overly simplistic for complex real-world applications, the classifier often performs surprisingly well, particularly for text classification tasks like spam detection or sentiment analysis. However, it is essential to understand its limitations. GNB can be sensitive to irrelevant features, and its performance might falter when feature independence is significantly violated. Nonetheless, its efficiency, paired with its ease of implementation, cements GNB as a robust starting point or baseline in numerous machine learning workflows.

### 2.3 Performance metrics

Within this study, diverse performance metrics are employed to compare models. These metrics encompass accuracy, recall, error rate, Youden's index, specificity, and F1 score.

#### 2.3.1 Accuracy

Accuracy pertains to the capacity of correctly classified data items, signifying the proximity of predictions to actual values. Mathematically, this can be articulated as:


(7) $$\begin{array}{l} \text{Accuracy }=\frac{\left(\text{TP }+\text{ TN}\right)\;}{\left(\text{TP }+\text{ FP }+\text{ TN }+\text{ FN}\right)} \end{array}$$

#### 2.3.2 Recall

Recall, often called the True Positive Rate or Sensitivity, is a vital evaluation metric in classification tasks. It gauges the model's capability to correctly identify positive instances out of all actual positive instances in the dataset. In essence, recall emphasizes the model's sensitivity to detecting instances of a specific class, which proves crucial in scenarios where missing even a single positive instance can have significant consequences. Mathematically, recall is calculated as the ratio of true positives (correctly predicted positive instances) to the sum of true positives and false negatives (positive instances wrongly classified as negatives). A high recall value indicates that the model is proficient in minimizing false negatives, thereby ensuring a higher level of coverage for the positive class. Recall assumes special importance when the cost of false negatives is considerably high. For instance, in medical diagnostics, failing to detect a serious condition can have severe repercussions. Thus, models that exhibit high recall can be paramount in such domains, even if they lead to a slightly higher number of false positives. Recall offers insights into a model's effectiveness in detecting positive instances, making it a crucial performance measure in applications where missing positive cases is undesirable. Balancing recall with other metrics like precision or F1-score helps create a comprehensive assessment of a classification model's capabilities and limitations.

#### 2.3.3 Youden's index

Youden's Index (Youdent) is generally formulated as:


(8) $$\begin{array}{l} \text{Youdent}=\text{max}\left\{\text{Sensitivity}\left(\text{a}\right)\;+\text{ Specificity}\left(\text{a}\right)\;-\;1\right\} \end{array}$$

The threshold that maximizes this index (a*) signifies the optimal threshold, as it balances the enhancement of biomarker discrimination by equally weighing specificity and sensitivity.

#### 2.3.4 Specificity

Specificity corresponds to the number of true negative values correctly recognized as negative. It is mathematically expressed as:


(9) $$\begin{array}{l} \text{Specificity}=\frac{\text{TN}}{\mathrm{TN}+\text{FP}} \end{array}$$

#### 2.3.5 F1 score

The F1 score blends precision and recall, striking a balance between them. The formula for calculating the F1 score is:


(10) $$\begin{array}{l} \text{F}1\text{ score}=\frac{2\;*\text{ Precision }*\text{ Recall }}{\left(\text{Precision }+\text{ Recall}\right)} \end{array}$$

The F1 score validates classifier efficacy by considering both precision and recall factors.

## 3 Results and discussion on model performance

In this section, we present the outcomes from various perspectives. We examine the performance of diverse machine learning techniques on the CKD dataset including XGC, NN, GNB, LR, RF, DT, KNN, and SVM. Our assessment covers three distinct scenarios: training and testing predictions set at 50, 75, and 90%. We evaluate predictive proficiency using six performance metrics: accuracy, error rate, recall, Youden's index, specificity, and F1 score. These metrics were evaluated across a total of one thousand runs.

**Figure 3:**
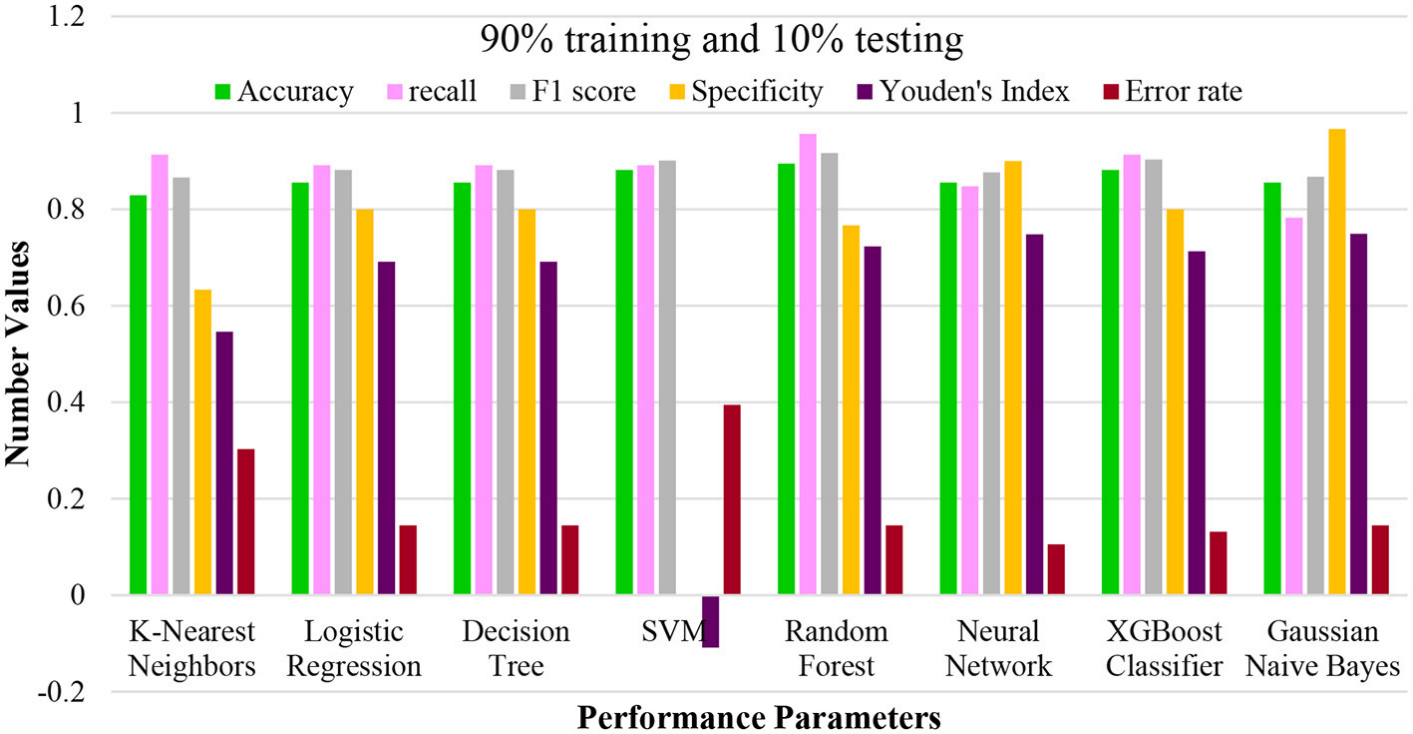
Performance plotting of techniques at 90% training and 10% testing ratio.

In the initial scenario, the training dataset constitutes 90%, while the remaining 10% is the testing dataset. The outcomes are presented in Figure 3. From the figure, it becomes evident that SVM and RF exhibit superior predictive abilities compared to the other models. The optimal predictive model achieved scores of 0.9171, 0.8671, 0.9484, 0.8155, 0.0643, 0.0829, and 0.9319 for mean accuracy, recall, sensitivity, Youden's index, error rate, and F1 score. RF ranks second among the models in terms of performance, while logistic regression display more robust performance compared to the remaining two models. To further substantiate the model superiority, we employed visualization tools.

In this evaluation, the machine learning techniques were rigorously tested using a 10% testing and 90% training data split. This approach aimed to provide a deeper understanding of their performance in situations with limited testing data, mirroring real-world scenarios with relatively more minor test sets. The results, as summarized in the provided table, underscore the capabilities of each technique under these circumstances. KNN exhibited an accuracy of 0.8289, suggesting its ability to classify instances with a considerable level of precision. Logistic regression and decision tree techniques maintained consistent performance, emphasizing their reliability across various scenarios. The SVM demonstrated strong recall and F1 score values, indicating its potential to identify positive instances even with a relatively constrained testing set. Notably, the random forest technique showcased remarkable performance, achieving an accuracy of 0.8947 and a high recall and F1 score, underscoring its robustness in handling complex classification tasks.

**Figure 4:**
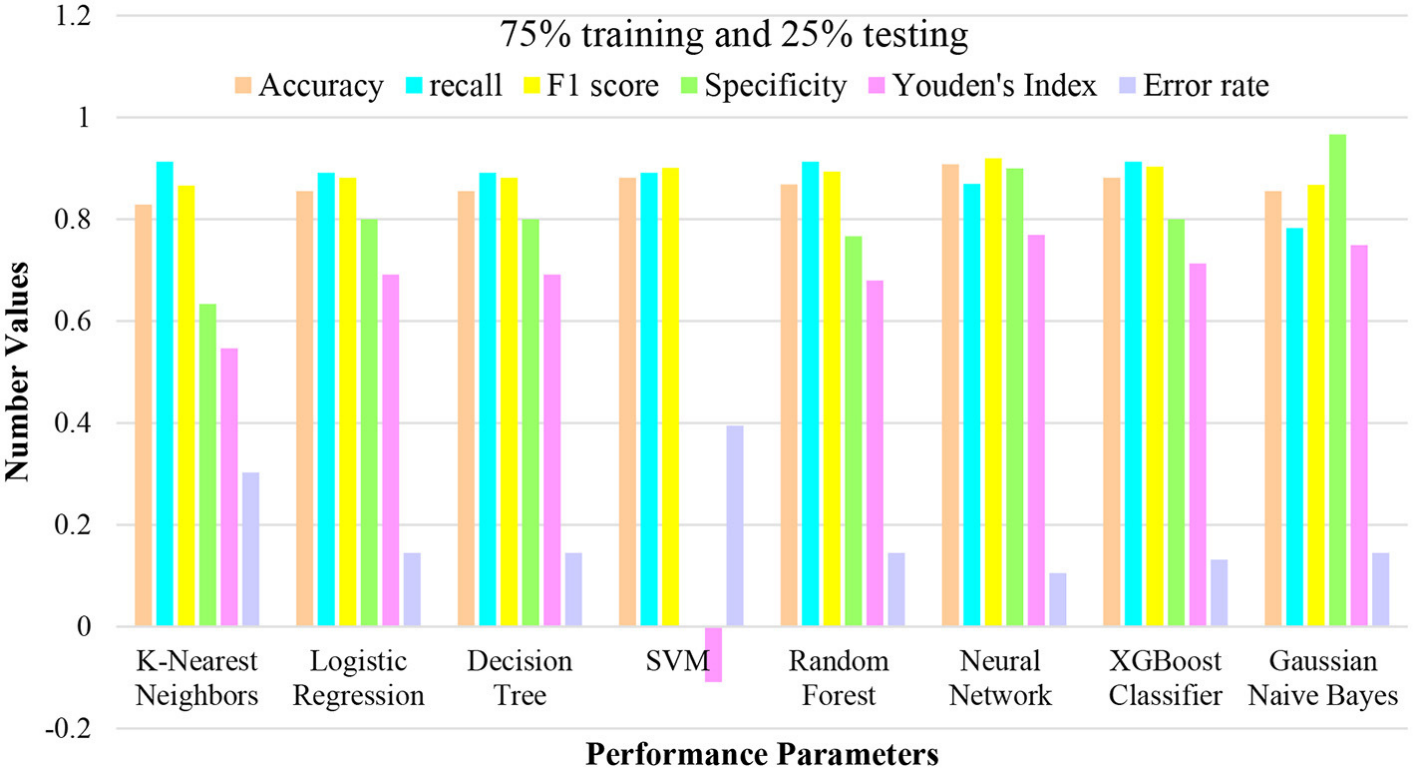
Performance plotting of techniques at 75% training and 25% testing ratio.

Furthermore, the neural network showcased its adaptability in this scenario, achieving competitive performance metrics. The XGC maintained its efficiency, with accuracy, recall, and F1 score values within the expected range. Lastly, the GNB technique displayed consistent accuracy, even with a smaller testing set. These outcomes reinforce the notion that machine learning techniques, when strategically employed and understood, can offer valuable insights even in scenarios with limited testing data. This evaluation using a 10% testing and 90% training split highlights the suitability of these techniques for various practical situations, providing stakeholders with insights to make informed decisions based on their specific requirements. For instance, Figure 4 graphically represents the mean accuracy, Brier score, sensitivity, Youden's index, specificity, and F1 score for all models.

In this analysis, a set of machine learning techniques was employed with a division of 25% of the data for testing and 75% for training. The objective was to evaluate the performance of each technique under these conditions and gain insights into their classification capabilities. The outcomes of these techniques are outlined in the provided. Notably, each technique was assessed based on performance metrics encompassing accuracy, recall, F1 score, specificity, Youden's Index, and error rate. These metrics collectively provide a comprehensive perspective on the effectiveness of each technique in both identifying positive instances and achieving overall classification accuracy. Among the techniques, KNN exhibited an accuracy of 0.8289, indicating its proficiency in correctly classifying instances in the given dataset. LR and DT techniques demonstrated comparable accuracy and recall values, emphasizing their suitability for this classification task.

**Figure 5:**
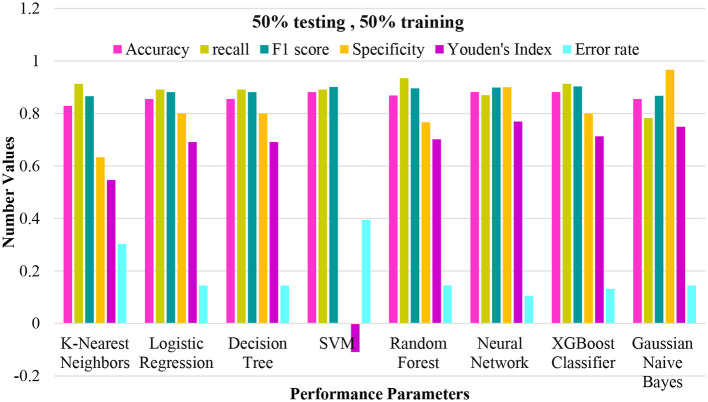
Performance plotting of techniques at 50% training and 50% testing ratio.

Additionally, the SVM technique showcased strong performance in recall and F1 score metrics, albeit with a trade-off in specificity. Furthermore, the analysis highlighted the Neural Network's impressive performance, achieving an accuracy of 0.9079, along with competitive recall and F1 score values. The XGC also displayed robust performance across several metrics, demonstrating its efficacy in classification tasks. This evaluation using a 25% testing and 75% training split sheds light on the relative strengths and limitations of each technique illustrated in Figure 5. Such insights provide valuable guidance for selecting an appropriate technique based on the specific requirements of similar classification scenarios.

The study employed a diverse range of machine learning predictive models, focusing on achieving accurate classification results for the dataset. The techniques utilized encompassed KNN, LR, DT, SVM with different kernel functions (linear, Laplacian, Bessel, and radial basis kernels), RF, NN, XGC, and GNB. The dataset was divided into a 50% training set and a 50% testing set to evaluate the performance of each technique. The obtained results are summarized in Figure 5. Notably, each technique was assessed based on multiple performance metrics, including accuracy, recall, F1 score, specificity, Youden's Index, and error rate. These metrics collectively offered a comprehensive view of each technique's effectiveness in positive instance identification and overall classification accuracy. For instance, KNN exhibited a strong recall rate of 0.9130, indicating its proficiency in identifying true positive instances. At the same time, logistic regression, decision tree, random forest, neural network, and XGC demonstrated competitive accuracy and recall values. It is noteworthy that SVM with various kernel functions exhibited distinct behavior regarding specificity and Youden's Index. This comparative analysis of different machine learning techniques contributes to a deeper understanding of their strengths and weaknesses in tackling the classification problem. Such insights are crucial for making informed decisions about the choice of technique when applying machine learning to similar classification tasks.

**Figure 6:**
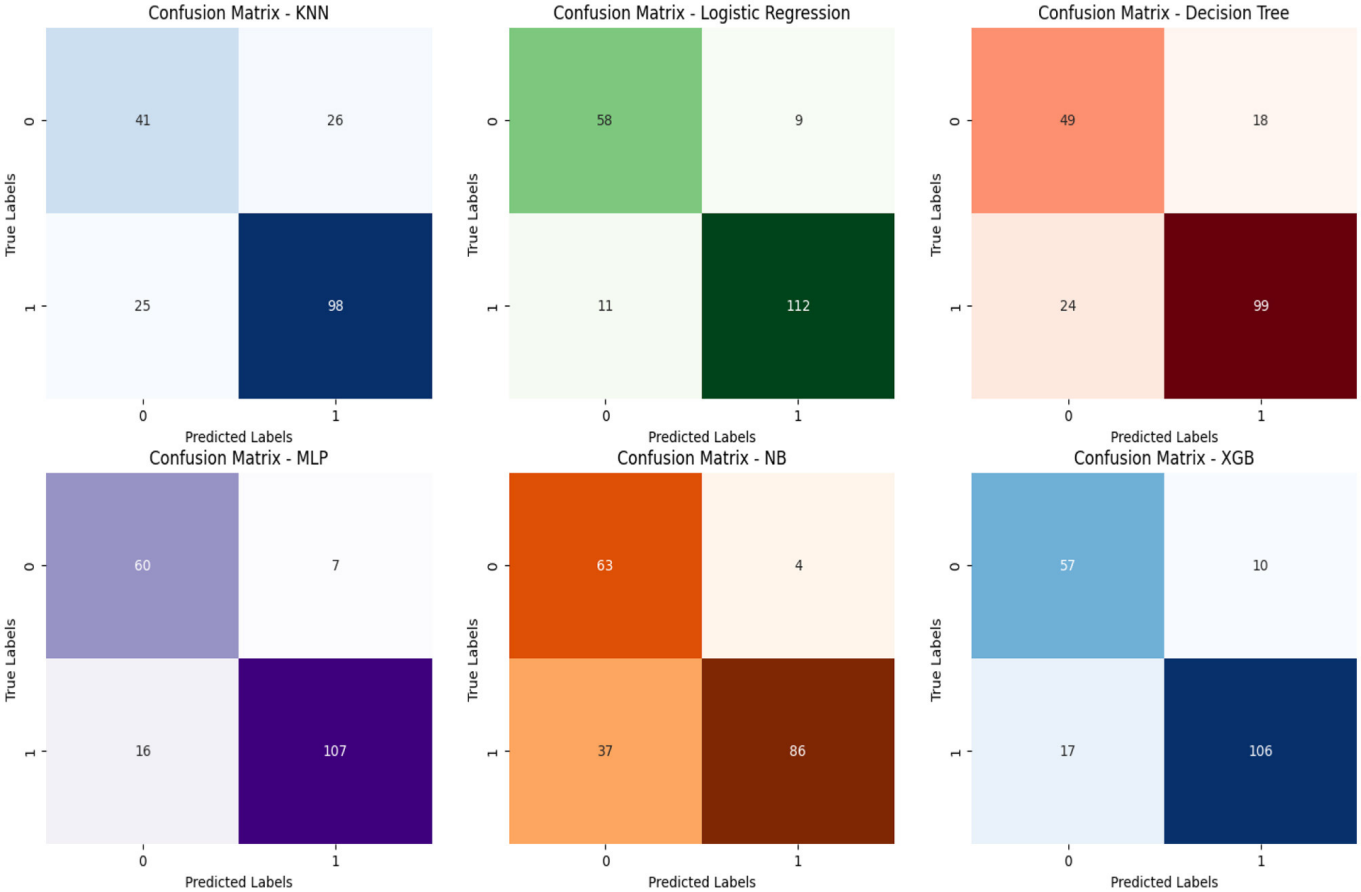
Confusion metrics of performance parameters.

**Figure 7:**
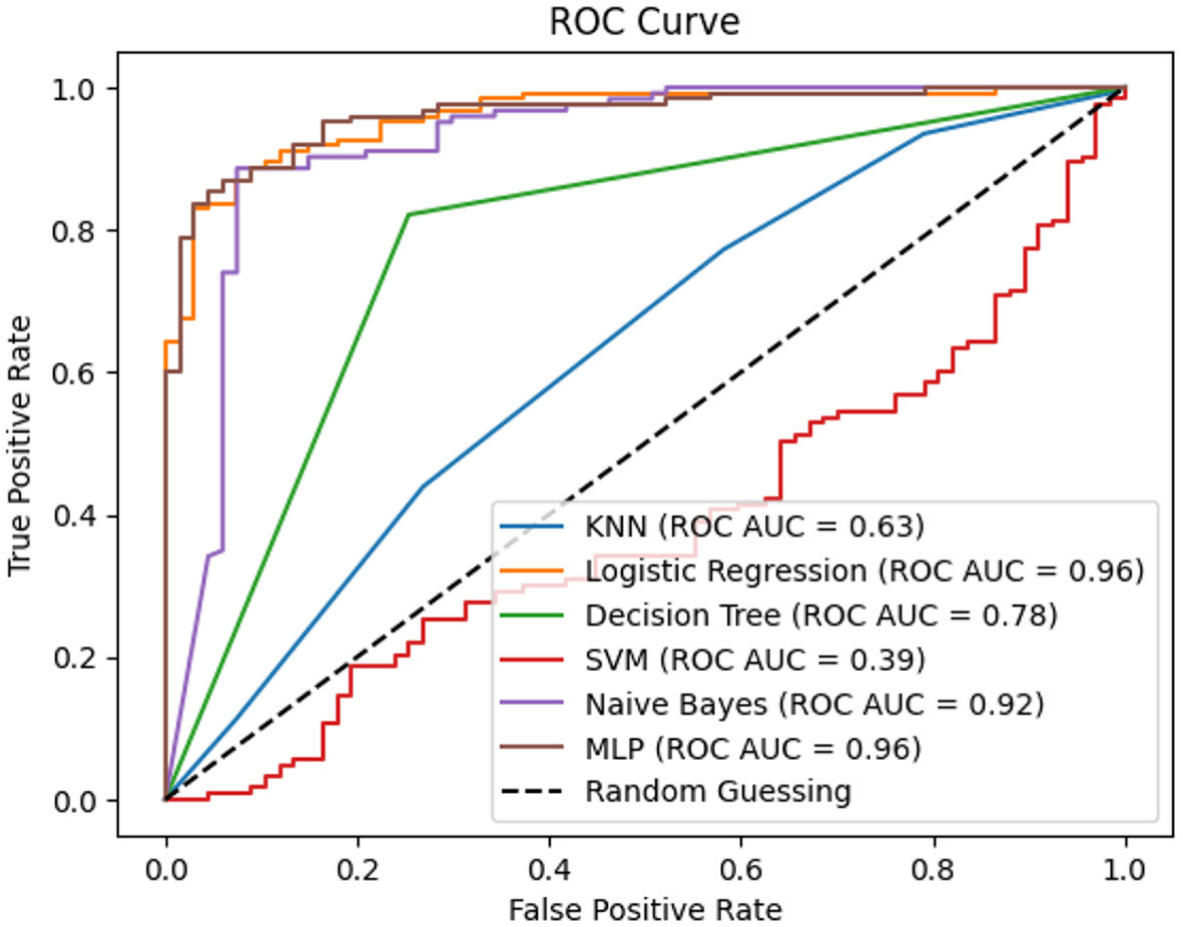
Receiver operating characteristic of performance parameters.

Figure 6 further enriches the analysis by incorporating confusion matrices. These matrices provide a comprehensive view of the classification performance of predictive models in the context of chronic kidney disease. By illustrating true positives, true negatives, false positives, and false negatives, the confusion matrices offer a tangible measure of model accuracy and effectiveness. This integrated approach combines the visualization of complete correlation with the quantification of classification outcomes, enabling a more holistic understanding of the intricate relationships between categorical attributes and their implications for disease classification and prediction. Figure 7 empowers stakeholders to make informed decisions by considering the underlying performance metrics of the predictive models.

In Figure 7, the integration of Receiver Operating Characteristic (ROC) curves enhances the analysis by presenting the performance of multiple classification techniques in the context of kidney chronic disease prediction. Each ROC curve corresponds to a specific classification technique: KNN, LR, DT, SVM, GNB, and MLP. The associated Area Under the Curve (AUC) values provide a quantifiable measure of each technique's ability to discriminate between positive and negative instances. The ROC AUC scores of the respective techniques showcase the diverse spectrum of their predictive capabilities. Notably, LR and MLP demonstrate remarkably high ROC AUC values of 0.96, indicating robust discriminatory power. DT follows suit with an AUC of 0.78, while GNB showcases competitive performance with an AUC of 0.92. KNN achieves an AUC of 0.63, positioning itself with moderate predictive strength. On the other hand, SVM exhibits a lower ROC AUC of 0.39, suggesting the need for further refinement. By presenting the ROC curves and their corresponding AUC values, Figure 6 offers an insightful comparison of each technique's ability to distinguish between positive and negative instances of chronic kidney disease. This visual and quantitative representation enables stakeholders to identify the most suitable technique for disease prediction, thereby aiding in clinical decision-making and shaping future research directions.

The comparative analysis of this study with recently published is shown in Table 2. All the previous studies used small dataset size with some models but this study emphasis on the large dataset size with most models.

**Table 2:** Results comparison with other studies.

**References**	**Method**	**Result**	**Drawback**
Zou and Liu (2021)	NN, RF, and KNN	Detection RF with 99.8% and F1 score	Small size of dataset
Nikhila (2021)	LR, SVM, ANN, and KNN	LR, SVM, ANN, and KNN with accuracy 97.5, 97.5, 65, and 66%	Small size of dataset
Poonia et al. (2022)	SVM and DT	SVM and DT with accuracy 96.75 and 91.75%	Small size of dataset
Salekin and Stankovic (2016)	ANN and RF	ANN and RF with accuracy 94.5 and 97.12%	Small size of dataset
This proposed study	XGC, NN, GNB, LR, RF, DT, KNN, and SVM along with predictive proficiency using six performance metrics	Greater accuracy with detection of error rate, recall, Youden's index, specificity and F1 score	Large size of dataset

## 4 Applications of ML and DL in medical field

**Figure 8:**
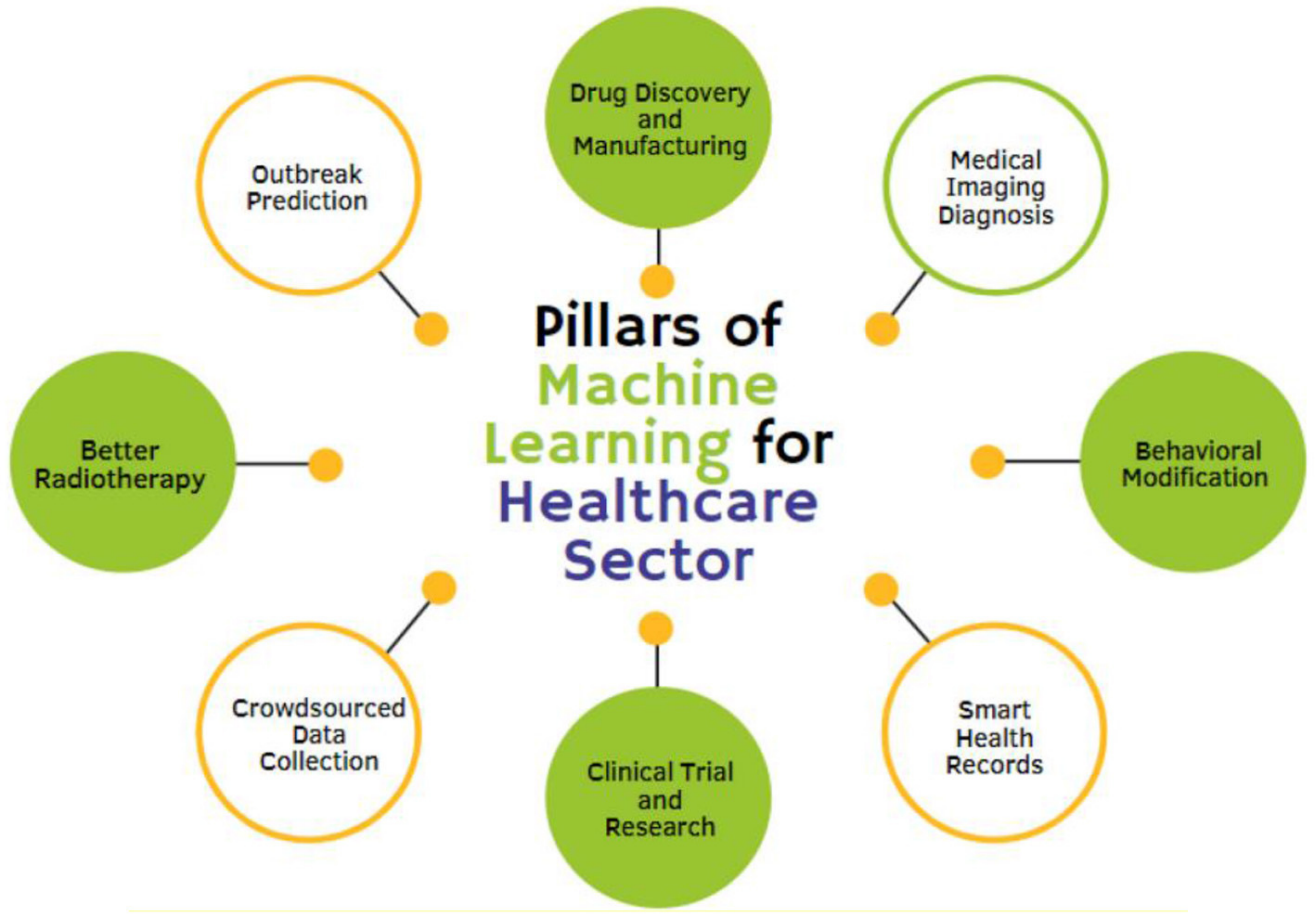
Applications of machine learning in healthcare sector.

ML and DL algorithms can be trained to analyze patient data, including lab results, medical history and imaging, to identify early signs of kidney disease. This enables timely diagnoses and personalized treatment plans, leading to better patient outcomes. ML and DL methods also predict the waste products such as creatinine and urea, as well as free water from the blood when the kidneys are in kidney failure. The detailed applications of ML in healthcare are presented in Figure 8.

## 5 Conclusions and future prospects

### 5.1 Comparative analysis of machine learning methods

This study presents a novel and comprehensive investigation into various machine learning techniques applied to a critical healthcare challenge. The study focuses on chronic kidney disease (CKD) and utilizes a dataset collected from district Buner in Khyber Pakhtunkhwa, Pakistan. What sets this research apart is its unique sourcing from an extensive case-control study involving CKD patients across the entire Buner district. This novel dataset provides a nuanced perspective on the disease's prevalence and characteristics within a specific geographic region. To address the complexity of CKD prediction, our research introduces an innovative approach by evaluating model performances across three distinct training and testing prediction scenarios: 50, 75, and 90%. This multi-scenario evaluation enhances the robustness of our findings, ensuring that the machine learning techniques' efficacy is thoroughly explored under varying data conditions. In the realm of classification analysis, we adopt a comprehensive set of performance metrics to assess model effectiveness holistically. These metrics include accuracy, error rate, recall, Youden's index, specificity, and F1 score. This thorough analysis goes beyond a simple comparison of accuracy and delves into nuanced aspects of model behavior, offering a comprehensive understanding of their strengths and weaknesses. The novelty of our research lies in its holistic approach, combining a unique dataset, multi-scenario evaluation, and a diverse set of performance metrics. By examining CKD prediction within a specific geographic context, our study contributes valuable insights to the field of healthcare machine learning. These insights are not only relevant to the Buner district but also hold the potential to inform healthcare strategies in other regions facing similar challenges.

### 5.2 Dominant model identification

Our findings unequivocally highlight the superiority of the SVM model across all three scenarios, underscoring its remarkable predictive prowess. Additionally, the RF model emerges as a strong contender regarding predictive capability. Furthermore, the DT test was employed to validate the dominance of predictive model accuracy measures, signifying the potential robustness of the identified models.

### 5.3 Future avenues and implications

This study lays a robust foundation for forthcoming medical research endeavors by extending its scope to predict the effectiveness of specific medications for various ailments. Moreover, the prominent machine learning models identified in this study hold the potential for predicting other medical conditions, such as heart disease, cancer, and tuberculosis. Furthermore, an opportunity exists to introduce a novel hybrid framework tailored to the same dataset, potentially yielding even more precise and efficient prediction outcomes. The implications of this research extend beyond the realm of chronic kidney disease prediction and offer promising avenues for advancements in medical prediction models and personalized treatment strategies.

## Data availability statement

The original contributions presented in the study are included in the article/supplementary material, further inquiries can be directed to the corresponding author.

## Ethics statement

The authors got ethical clearance for this study from the management of medical complex in Buner, Khyber Pakhtunkhwa, Pakistan.

## Author contributions

NK: Conceptualization, Data curation, Formal analysis, Writing — original draft. MR: Investigation, Methodology, Software, Writing — original draft, Writing — review & editing. NM: Data curation, Formal analysis, Methodology, Resources, Supervision, Visualization, Writing — original draft, Writing — review & editing. NB: Formal analysis, Software, Validation, Visualization, Writing — review & editing. GA: Conceptualization, Data curation, Formal analysis, Investigation, Software, Writing — original draft, Writing — review & editing. AY: Conceptualization, Funding acquisition, Project administration, Supervision, Writing — original draft, Writing — review & editing. ET: Conceptualization, Funding acquisition, Project administration, Supervision, Writing — original draft, Writing — review & editing.
