# Corrigendum: Unveiling the predictive power: a comprehensive study of machine learning model for anticipating chronic kidney disease

**DOI:** 10.3389/frai.2024.1373254

**Published:** 2024-02-02

**Authors:** Nitasha Khan, Muhammad Amir Raza, Nayyar Hussain Mirjat, Neelam Balouch, Ghulam Abbas, Amr Yousef, Ezzeddine Touti

**Affiliations:** ^1^Department of Electrical Engineering, Nazeer Hussain University, Karachi, Pakistan; ^2^Department of Electrical Engineering, Mehran University of Engineering and Technology, Khairpur Mirs, Sindh, Pakistan; ^3^Department of Electrical Engineering, Mehran University of Engineering and Technology, Jamshoro, Sindh, Pakistan; ^4^Department of Zoology, Shah Abdul Latif University Khairpur Mirs, Khairpur Mirs, Pakistan; ^5^School of Electrical Engineering, Southeast University, Nanjing, China; ^6^Electrical Engineering Department, University of Business and Technology, Jeddah, Saudi Arabia; ^7^Engineering Mathematics Department, Alexandria University, Alexandria, Egypt; ^8^Department of Electrical Engineering, College of Engineering, Northern Border University, Arar, Saudi Arabia

**Keywords:** forecasting, public health, medicine, deep learning, machine learning

In the published article, there was an error in the Acknowledgments, which listed an incorrect project number (“NBU-FFR-2023-0177”).

The correct Acknowledgments appear below.

